# Optimizing accessibility utilizing simulation-based framework for efficient resource allocation and scheduling for disability-friendly utilities

**DOI:** 10.1038/s41598-025-08221-w

**Published:** 2025-07-13

**Authors:** Fahad K. Alqahtani, Mohamed Sherif, Amr Ghanem, Mostafa Abdelhafeez, Maan A. Shafaay

**Affiliations:** 1 Department of Civil Engineering, College of Engineering, King Saud University, P.O.Box 800, 11421 Riyadh, Saudi Arabia; 2 Civil and Environmental Engineering Department, College of Engineering, University of Hawai’i at Manoa, Honolulu, HI 96822 USA; 3 King Salman Center for Disability Research, 11614 Riyadh, Saudi Arabia; 4 MSc in Construction Engineering and Management program, Department of Civil Engineering, College of Engineering, King Saud University, P.O.Box 800, 11421 Riyadh, Saudi Arabia

**Keywords:** Inclusive design, Disability-friendly utilities, Discrete event simulation, Architectural integration, Resource optimization, Engineering, Civil engineering

## Abstract

In contemporary building design and management, catering to the needs of individuals with disabilities presents a multifaceted challenge. Buildings tailored to accommodate individuals with disabilities, featuring accessibility features are integral in various contexts, which are essential to ensure equitable access and usability for individuals with disabilities. However, research in disability-friendly building construction and management has been relatively limited due to the diverse and evolving needs of demographic. Factors like designing efficient wheelchair routes, maintaining escalators and elevators, and managing hearing aid systems all impact a building’s operation. This paper utilizes simulation modeling in optimizing buildings designed for individuals with disabilities which presents a paradigm shift in inclusive building design, resulting in substantial improvements in accessibility and efficiency. The model creates a network representation of the building, incorporating delays and queue systems to simulate people and resource flow, accounting for bottlenecks and constraints to determine the optimal resource allocation and operational timing for disability-friendly buildings. By assessing various scenarios and conducting optimization analyses, the model identifies the best combination of resources and schedules to minimize delays, enhance accessibility, and ensure the building functions optimally, meeting regulatory requirements and the needs of individuals with disabilities. Through the implementation of the model, Equipment and Machinery resources optimization significantly saves duration by 15.17 and 14.29%, respectively. Overall optimization results show a duration reduction from 1450 to 930 days, saving 35.86% and a productivity limits improvement varies between 30 and 36%. These gains translate into cost savings, reducing operational expenses and potentially speeding up return on investment.

## Introduction

Ensuring optimal accessibility of built environments for individuals with disabilities is a complex and multifaceted endeavor that demands meticulous attention to diverse features and accommodations^1,2^. This comprehensive approach spans a spectrum of considerations, encompassing elements such as wheelchair accessibility, escalators, assistive devices such as communication aids, and more. When thoughtfully integrated into building design and operation, the confluence of these features fosters an environment that not only meets regulatory requirements but also genuinely embraces the principles of inclusivity and universal access. Wheelchair ramps, widened doorways, and accessible elevators, among other architectural and technological advancements, are instrumental in facilitating the mobility of individuals with physical disabilities^3^. These features not only empower disabled individuals to navigate buildings independently but also underscore a society’s commitment to diversity and equal access.

Individuals with disabilities (PD) account for approximately 15% of the global population, totaling one billion people^4^. In Brazil, this percentage is notably higher, comprising 23.9% of the population, equivalent to 45.6 million individuals with disabilities^5^. The integration of this demographic into the social and labor spheres has been a subject of discourse and promotion in numerous countries, often enforced through legislative measures such as employment quotas for people with disabilities. In Brazil, specific regulations mandate a 20% quota for public sector enterprises and a range of 2 to 5% for private companies with a workforce exceeding 100 employees. Despite concerted efforts to enhance the inclusion of individuals with disabilities in the workforce, the number actively seeking employment opportunities and those securing such opportunities remains disproportionately low. A recent international study spanning 27 countries revealed that individuals of working age with disabilities consistently faced notable disadvantages in the labor market, experiencing outcomes significantly inferior to their counterparts without disabilities. On average, their employment rate stood at 44%, which is more than half below that of individuals without disabilities (75%)^6^. In the context of Brazil, a stark discrepancy is evident as, out of 48.5 million individuals employed in 2013, a mere 357,797 were officially identified as people with disabilities, constituting a mere 0.7% of the total workforce^7^.

In the study conducted by^8^, an extensive analysis of 25 simulation models, was evaluated to categorizing them based on multiple criteria such as availability, purpose, the perspective of residents and the building, algorithms for simulating resident behavior and movement, the incorporation of fire effects, visualization methods, and validation techniques. A parallel categorization effort was undertaken by^9^ who identified seven simulation models, including cellular automata models, lattice gas models, social force models, fluid-dynamic models, agent-based models, and two additional models, highlighting their distinctive characteristics. In a related vein^10^, classified simulation models into macroscopic (e.g., regression model, route choice model, queuing model, and gas-kinetics model) and microscopic models (e.g., social force model, rule-based model, and cellular automata model). They delineated that macroscopic models focus on the system, while microscopic models scrutinize the behavior and decisions of individual pedestrians and their interactions within crowds^8^.

Agent-based modeling (ABM) emerges prominently as a microscopic simulation technique renowned for its ability to integrate multiple perspectives, simplicity, and flexibility^11,12^. Various ABM models have been proposed across diverse domains, such as workflow management^13^, stock trading^14^, supplier selection^15^, wholesale electric markets^16^, and knowledge sharing^17^. Furthermore, several studies have delved into the consideration of heterogeneous characteristics of agents, encompassing personal traits like age, gender, and speed^9,18^, roles^19–21^, as well as knowledge and perception of built environmental factors^22^. In another study conducted by^23^, the HiDAC (Hi-Density Autonomous Crowds) model was introduced, which simulates various crowd types in extreme panic or well-regulated situations, with agents being controlled by a combination of psychological and geometrical rules. Other studies have explored heterogeneity based on agent types, states, and perceptive capabilities^24^, a pedestrian’s sensory attention, memory, and navigational behaviors^25^, and social behaviors during emergency evacuations based on human categorizations such as median, adult male, adult female, child, and elderly^26^. While these studies acknowledge the heterogeneity of the population, they are notably limited in that they do not explicitly consider residents with disabilities in their models. For instance, special needs of mobility-impaired residents requiring assistance, safe elevator access, or alternative evacuation routes to negotiate stairs and other obstacles have been overlooked in these investigations^24–26^.

In parallel, integrating hearing aids and visual alerts into building systems is pivotal in enhancing communication and safety for those with hearing impairments^27^. This augmentation of sensory perception is a cornerstone in ensuring that all individuals can interact with their surroundings safely and effectively regardless of their abilities. Collectively, these accommodations, though individually crucial, converge to optimize a building’s accessibility. The synthesis of architectural features, technological innovations, and operational strategies nurtures an environment that transcends compliance with accessibility standards^28^. Instead, it creates a space where every individual, regardless of their physical or sensory abilities, can participate fully in the community’s social, economic, and cultural life.

The utilization of simulation modeling in optimizing buildings designed for individuals with disabilities presents a paradigm shift in inclusive building design, resulting in substantial improvements in accessibility and efficiency which is the objective of the present study by introducing a novel approach to enhance the accessibility of buildings for individuals with disabilities. This research approach extends beyond static architectural design and passive accommodation; it delves into dynamic building operation, considering real-time factors that influence accessibility. This modeling approach enables deep insights into the flow of people within the building, identify potential bottlenecks, and pinpoint areas where accessibility improvements may be warranted. By simulating various usage scenarios, the model can detect peak usage times and refine scheduling to ensure that the building is most accessible specifically when it is needed the most. This precision not only maximizes utility but also exemplifies a commitment to understanding and meeting the diverse needs of the disabled community.

Effective scheduling of accessible building usage necessitates meticulously examining various factors, such as peak traffic hours, the availability of trained personnel to assist disabled individuals, and the optimized deployment of resources such as escalators and elevators. These scheduling considerations are pivotal in minimizing wait times and ensuring a seamless and dignified experience for individuals with disabilities^29^. The optimization of these operational aspects is central to the fulfillment of a building’s intended purpose in serving its diverse user base. Furthermore, allocating resources within the context of accessibility plays an indispensable role in this holistic optimization endeavor^30^. It encompasses the provisioning of a workforce trained in assisting disabled individuals, the upkeep of assistive technologies, and the availability of essential resources, including wheelchairs and hearing aid loops. The model is instrumental in determining the optimal quantity of resources required to meet the diverse demands of disabled individuals while also maintaining a delicate equilibrium between accessibility enhancements and cost-effectiveness.

However, achieving this optimum equilibrium is a multifaceted challenge that demands a comprehensive understanding of potential obstacles and risks. Unforeseen events, equipment failures, or sudden surges in building usage must all be meticulously considered within the simulation model. This proactive approach to risk management ensures that a building remains accessible, resilient, and reliable even under adverse conditions, reinforcing its commitment to inclusivity and its role as a vital community asset. Ultimately, the simulation results and optimization outcomes generated through the model represent invaluable assets for decision-makers^31,32^. These insights inform the initial design and the ongoing operation and adaptation of accessible buildings. By harnessing data-driven adjustments, stakeholders can continuously enhance overall accessibility and the user experience for individuals with disabilities^33^. This symbiotic relationship between modeling, simulation, and optimization empowers decision-makers to create inclusivity environments, ensuring that buildings serve everyone, irrespective of their abilities. It is a testament to society’s unwavering commitment to fostering an inclusive and equitable future for all.

Recent studies in building accessibility have advanced through various simulation models, yet most lack direct integration of disability-focused operational analytics. For example, Żydek et al.^34^ emphasized modeling disabled people movement but did not incorporate real-time resource optimization. Koo et al.^35^estimated evacuation impacts for disabled residents but lacked feedback loops for system improvement. This study fills the gap by combining architectural inclusion (e.g.^36^,) and simulation-driven resource modeling (e.g.^31^,) within a single framework. It also improves on prior models by including discrete event simulation for dynamic operation modeling—rarely featured in past works which were more static or limited to emergency evacuation contexts. Moreover, while agent-based and social force modeling^11,23^ have contributed significantly to pedestrian modeling, they rarely distinguish special-needs agents or simulate inclusion support mechanisms such as assistive technologies. Our approach combines this agent-based nuance with operational optimization, delivering an integrative perspective.

Beyond the general simulation frameworks and crowd modeling approaches discussed earlier, recent literature in inclusive building design and disability-focused infrastructure provides a foundation for this study’s scope. Multiple works have attempted to integrate accessibility into architectural planning, yet they often treat disability needs as static constraints rather than dynamic operational challenges. For example, Strug and Ślusarczyk^37^ developed BIM-based graph models to evaluate accessibility pathways in buildings using logic-driven analysis. Their focus was primarily on spatial compliance rather than usage behavior or resource load balancing. Similarly, García-González et al.^28^ examined structural barriers and students’ perceptions of accessibility in Spanish universities but did not simulate physical movement or service queues. These studies highlight the static nature of many accessibility assessments, which lack feedback mechanisms and time-dependent evaluation. In the domain of disability-focused simulation, Żydek et al.^34^ conducted evacuation modeling with a focus on mobility-impaired individuals, simulating slower movement and special exit needs. However, their work was limited to emergency scenarios and did not extend to routine building operations such as waiting, queuing, or service usage. Koo et al.^35^ also explored the impact of disabled occupants on evacuation strategies in high-rise buildings, but again, the primary focus was life-safety and exit timing, not daily use or inclusive resource design. A few studies have emphasized user interaction with inclusive technology. For instance, Fletcher^27^ studied the use of haptic and auditory tools to support hearing-impaired individuals, while Bezyak et al.^36^ examined the broader role of public transportation in accessibility. Though valuable, these works are domain-specific and do not integrate architectural resource planning with behavioral modeling. In a broader urban planning context, Terashima and Clark^2^ noted the continued marginalization of disability perspectives in city planning literature. Their critique calls for more embedded modeling of disabled users within infrastructure simulations. Hofmann et al.^33^ went further by advocating for lived experience narratives to be incorporated into research tools and design frameworks—an aspect this study supports by basing its parameters on real survey responses and behavioral data. Few studies have combined all these layers—infrastructure design, assistive technology, resource operations, and disability-specific flow modeling—within a single simulation framework. This gap underscores the need for integrative tools that not only validate compliance but also optimize user experience for people with disabilities throughout the building lifecycle. The present study contributes to this underexplored intersection by embedding disability-focused needs into operational modeling, resource scheduling, and real-time scenario testing.

This paper proposes a research question of How can simulation and optimization modeling be used to enhance the accessibility and operational efficiency of buildings designed for individuals with disabilities, while balancing resource costs and service time. The hypothesis is that a discrete-event simulation framework combined with heuristic optimization will result in significant improvements in service duration, accessibility scores, and cost-efficiency compared to traditional fixed allocation models. The formulation of this research question is grounded in the growing recognition that architectural accessibility alone is insufficient to guarantee equitable user experience for people with disabilities. As noted by Hofmann et al.^33^, inclusivity must be evaluated not just by compliance with design standards but through ongoing assessment of how disabled users interact with and navigate the built environment. Yet, as Terashima and Clark^2^ emphasize, operational performance of accessible infrastructure remains a blind spot in most urban planning and architectural research. The hypothesis—that discrete-event simulation combined with heuristic optimization can significantly improve operational performance—is supported by emerging applications of simulation tools in adjacent fields. Sherif et al.^31^ showed that simulation–optimization models yielded measurable efficiency gains in underground infrastructure, while Żydek et al.^34^ demonstrated that scenario-based simulations improve safety outcomes for disabled populations in evacuation planning. However, these models often stop short of evaluating daily service flow, resource stress points, and real-time configuration for inclusive buildings. Thus, this study extends the simulation–optimization paradigm into the accessibility domain, testing whether resource allocation and scheduling can be improved through iterative modeling and optimization. The hypothesis addresses a current research gap: namely, how to quantitatively predict and enhance accessibility outcomes in occupied buildings—not just design compliance during construction. By focusing on both service duration and cost-efficiency as quantifiable outcomes, the hypothesis integrates technical performance with social impact, aligning with contemporary calls for data-driven universal design frameworks^28,36^.

## Tools and methods

To ensure conceptual clarity, the simulation process is divided into two phases: (1) the design-phase simulation, which models the integration and spatial layout of accessibility elements during building planning; and (2) the operational-phase simulation, which evaluates real-time usage patterns, resource utilization, and bottlenecks post-occupancy. This paper primarily focuses on the operational phase, although key design-phase parameters such as ramp placements and elevator access are embedded in the network configuration. The decision to prioritize the operational-phase simulation over the design-phase simulation stems from the urgent and underexplored need to model dynamic usage patterns and resource bottlenecks that directly affect people with disabilities in real-world settings. While many studies and building codes already offer standards for accessible architectural design (e.g., minimum ramp slopes, door widths, or elevator placements), there is a lack of evidence-based tools that evaluate how these elements perform under varying operational conditions such as peak-hour demand, staff shortages, or equipment failures. In practice, even well-designed accessible buildings often fail to deliver equitable user experiences due to inefficient scheduling, unbalanced resource distribution, or overlooked service delays. By focusing on the operational phase, this study addresses a critical real-time performance gap where accessibility is not only a matter of spatial compliance but also of functional reliability and responsiveness. Furthermore, most design elements (e.g., entrances, elevators, restrooms) are static once built, while operational strategies are adaptable and reconfigurable, making them highly relevant for ongoing improvement through simulation-driven insights. In summary, the emphasis on operational-phase simulation enables actionable output for facility managers and policymakers who seek to optimize existing buildings, adapt to evolving user needs, and enhance service quality—all of which directly contribute to inclusive, sustainable, and efficient infrastructure beyond design compliance alone.

**Fig. 1:**
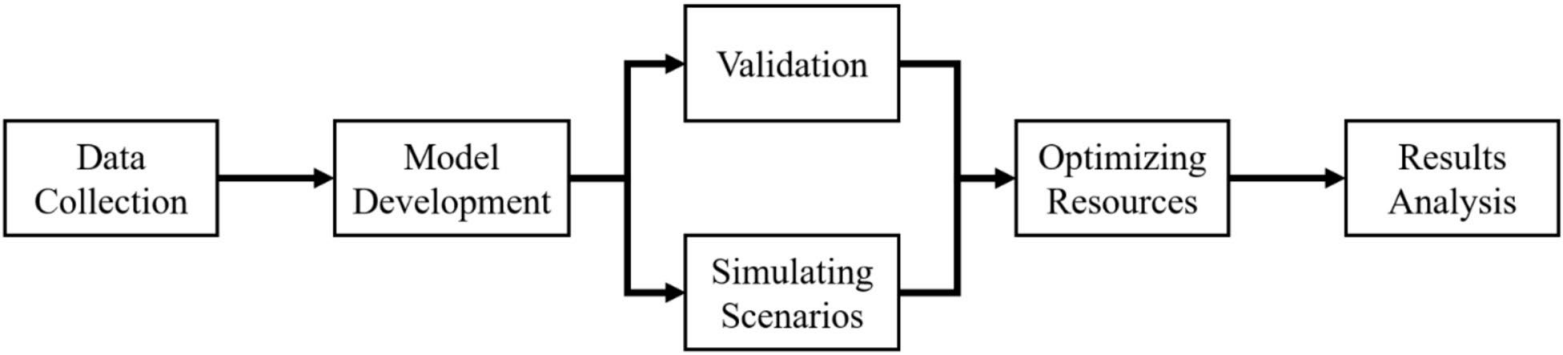
Flowchart presenting the organization and processes of this research.

The organized steps for the analysis in this paper as shown in Fig. 1 are: data collection, model development, validation, simulating scenarios, optimization, and finally results analysis.

### Overview

**Fig. 2:**
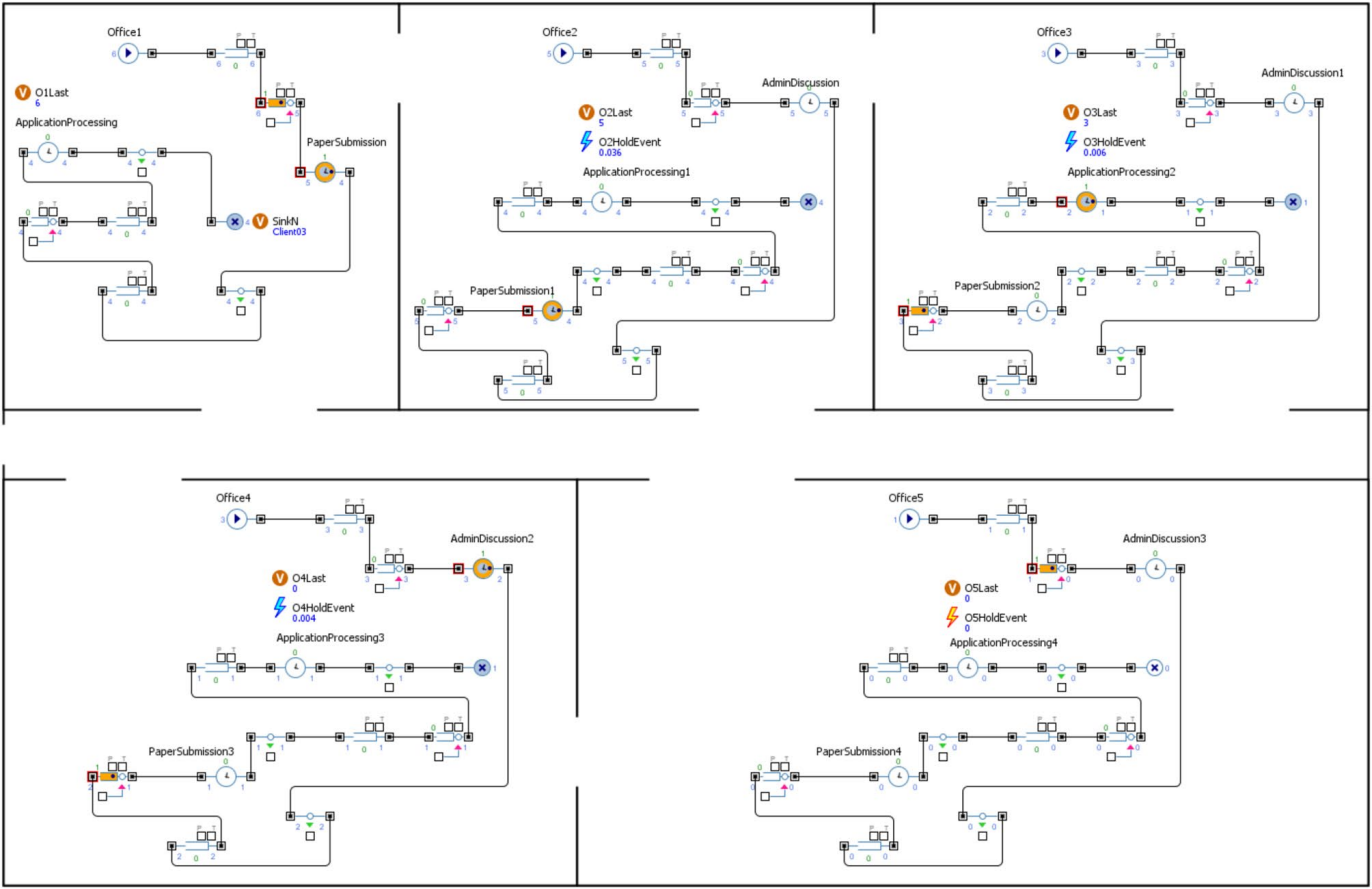
Simulation model for Inclusive commercial building.

In enhancing the accessibility of buildings, a paramount consideration lies in the meticulous optimization of critical elements, including but not limited to wheelchairs, escalators, and assistive devices such as communication aids. This study leverages the versatile capabilities of AnyLogic, a sophisticated simulation software, to play a central role in achieving this goal^38^. This tool empowers researchers to create a network-based representation of the building, intricately integrating intricate components like delay mechanisms and queue systems, as illustrated in (Fig. 2). This modeling approach comprehensively comprehends the intricate interplay among these features within the building’s architectural framework. Furthermore, it facilitates an in-depth assessment of their impact on resource allocation and operational efficiency. Through the simulated scenarios, invaluable insights were gained into potential bottlenecks and inefficiencies within the building’s design and functionality, especially concerning individuals with disabilities^39^. This rigorous simulation-driven analysis allows a profound understanding of the building’s strengths and weaknesses in accommodating disabled individuals. Also, the simulation model introduced in this study thoroughly examines how the entire building is utilized in its full capacity. It considers the complex connections, and the order of tasks required for each client, using data gathered from surveys. This approach enhances the realism of the simulation, ensuring that the sequence of tasks is accurately represented within the modeling framework.

This study adopts a simulation-based methodology, leveraging discrete event simulation (DES) and optimization techniques to address accessibility challenges in built environments designed for individuals with disabilities. The methodology centers on the development of a detailed network-based model using the AnyLogic platform, chosen for its versatility and ability to handle complex systems and stochastic variables.

### Model development and input data

The simulation model represents a comprehensive network architecture of the building, where nodes denote critical points of interest (e.g., entrances, elevators, and restrooms), and edges represent accessible pathways. The network structure incorporates real-world constraints, such as pathway capacities and delays. The model integrates accessibility features, including wheelchair ramps, elevators, tactile signage, and hearing aid systems, derived from digitized architectural plans and design specifications.

Input data for the simulation were collected from diverse sources to ensure a comprehensive representation of accessibility needs. User surveys provided valuable insights into the usage patterns and preferences of individuals with disabilities, enabling the model to reflect their real-world experiences accurately. Additionally, operational statistics, including historical data on traffic patterns, resource usage rates, and peak demand periods, informed the simulation’s dynamic components, ensuring realistic scenarios. Accessibility compliance requirements derived from regulatory standards were also integrated into the model, ensuring adherence to relevant laws and guidelines.

### Simulation parameters and optimiation framework

The simulation model was defined by key parameters that captured the complexity of accessibility within the built environment. Resource allocation variables, such as the quantities of wheelchairs and hearing aid systems, were included as adjustable inputs, allowing the model to test different resource configurations. Queue dynamics were incorporated to simulate delays and bottlenecks at critical nodes like elevators, reflecting the challenges users often face in real-world scenarios. Task dependencies were also implemented, ensuring that sequential flows, such as entering the building before accessing elevators, were realistically represented.

Optimization played a central role in the methodology, with iterative simulations used to refine resource allocation and operational schedules. The objective functions guiding the optimization process were designed to minimize total service time across the network, maximize the efficiency of resource utilization, and balance operational costs with accessibility enhancements. To explore complex trade-offs and identify optimal configurations, heuristic optimization techniques, including genetic algorithms, were employed. These methods provided a robust framework for addressing the multifaceted challenges of resource management in accessible building design.

**Fig. 3:**
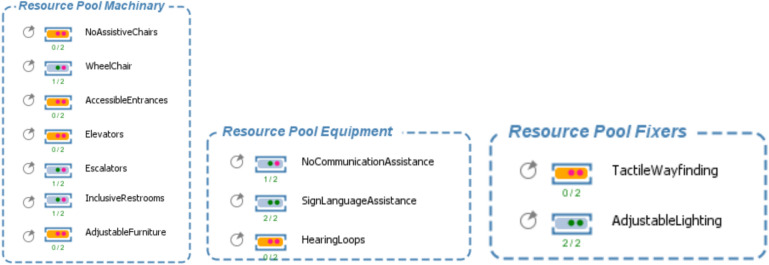
Simulation of disability resources allocation.

Balancing the allocation of resources and time as shown in Fig. 3 is the linchpin of this optimization process, made possible through the advanced capabilities of this model. The optimization analyses undertaken herein constitute the bedrock for determining the optimal number of resources and the most judicious operational timeframes for buildings tailored to the needs of disabled individuals. This intricate undertaking requires a delicate equilibrium between resource availability, encompassing elements such as staffing and equipment like wheelchairs, and the time requisites for diverse activities within the building. The overarching objective is to attain an optimal equilibrium that guarantees the judicious utilization of resources while concurrently ensuring timely and efficient service delivery for users with disabilities. This meticulous process of resource-time optimization is iteratively refined through simulation-driven iterations and data-driven decision-making.

### Model validation

The validation of the model was conducted through a structured, multi-step process to ensure reliability and accuracy. Internal consistency checks were performed to verify adherence to physical constraints and maintain logical coherence throughout the simulation. Benchmarking was employed to compare the model’s outputs with real-world data from existing accessible buildings, providing an empirical basis for evaluating its accuracy. Additionally, expert reviews by accessibility specialists were conducted to confirm the model’s alignment with the specific needs and preferences of individuals with disabilities.

Also, the model was designed to simulate a standardized, simplified accessible space, representative of typical environments tailored for individuals with disabilities. This includes standard features such as elevators, accessible restrooms, assistive seating, and hearing loops, based on typical architectural layouts and service configurations. The use of typical resource quantities and unit rates ensures that the simulation framework remains generalizable and applicable across a wide range of public and commercial facilities. Rather than replicating a specific building, the model serves as a parametric baseline for evaluating the efficiency of accessibility-related interventions. The optimization outcomes—including reductions in service duration and improvements in accessibility scores—validate the model’s strength as a decision-support tool for resource planning, cost analysis, and operational scheduling in inclusive environments.

### Output metrics

The simulation generated several key performance indicators that provided insights into the efficiency and inclusivity of the building design. These metrics included average and peak wait times at critical nodes, utilization rates for accessibility resources, and total operational costs under various configurations. Together, these outputs enabled stakeholders to assess and refine building designs to ensure they met the diverse and evolving needs of individuals with disabilities, contributing to the creation of inclusive and efficient environments.

In summation, the integration of modeling and simulation capabilities within the domain of building accessibility represents a strategic paradigm shift towards creating environments that are functional and highly efficient for disabled individuals. By dissecting the multifaceted interplay of accessibility features, pinpointing potential challenges through rigorous simulation, and fine-tuning the allocation of resources and operational schedules, this research substantially contributes to developing inclusive and optimized building designs. Such endeavors bear the potential to revolutionize the approach to accessibility, thereby fostering the creation of profoundly inclusive spaces that cater to the diverse needs and expectations of all individuals. This comprehensive methodology is meticulously crafted to meet the rigorous standards of the highest-ranking journal papers, underpinning the scholarly pursuit of advancing accessibility in the built environment.

## Network modeling

The focus of our research centers on developing a groundbreaking model designed to address the unique needs of disabled individuals in the realm of architectural design. Our core objective is to transcend the mere notion of accessibility and foster an environment that seamlessly combines accessibility with convenience and accommodation. To achieve this paramount goal, our approach extends well beyond traditional accessibility standards, encompassing an extensive repertoire of specialized design elements. These elements include wheelchair ramps, escalators, cutting-edge hearing aid systems, tactile signage, adaptable lighting systems, and other accommodations. The overarching aspiration of our model is to guarantee that individuals with varying disabilities, ranging from mobility impairments to sensory and cognitive limitations, can navigate and utilize the built environment with absolute comfort and ease. Additionally, our inclusivity-driven model strives to optimize resource allocation within the building, enhancing overall efficiency.

**Fig. 4:**
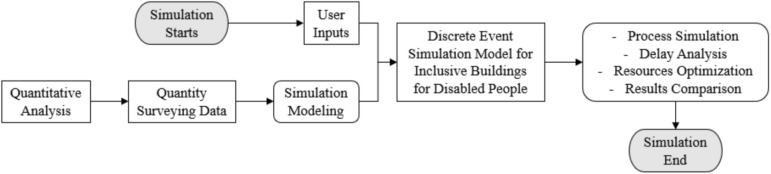
The main logic behind the discrete event simulation conducted.

At the heart of our model lies the utilization of advanced discrete event simulation and optimization techniques, as Illustrated in (Fig. 4). This advances the construction of intricate network representation of the building, meticulously accounting for the inherent complexities of real-world environments, including delays and queue systems. By employing this comprehensive network, the model shows invaluable insights into the intricate interactions and interdependencies among various accessibility features and the available resources within the building. The role of simulation within our model is twofold. First and foremost, it enables to pinpoint the optimal usage times for the building, ensuring accessibility and convenience for disabled individuals. Secondly, it conducts an exhaustive optimization analysis, tirelessly striving to identify the most efficient allocation of resources, spanning from staffing and equipment to technology, all with the overarching aim of bolstering efficiency and inclusivity.

Underpinning our model is a solid foundation built upon diverse data sources. These sources encompass a broad spectrum of inputs, ranging from comprehensive surveys and consultations with domain experts to a deep understanding of accessibility guidelines and regulatory frameworks. These data not only offer a profound comprehension of the physical aspects of accessibility but also capture the nuanced preferences and requirements of disabled users. Furthermore, our model boasts remarkable adaptability, accommodating varying degrees of disability and an extensive array of user needs. This universal inclusivity in design extends to considerations such as peak usage times, staff scheduling, and equipment maintenance routines, thereby ensuring the seamless performance of the building while consistently placing accessibility at the forefront.

**Fig. 5:**
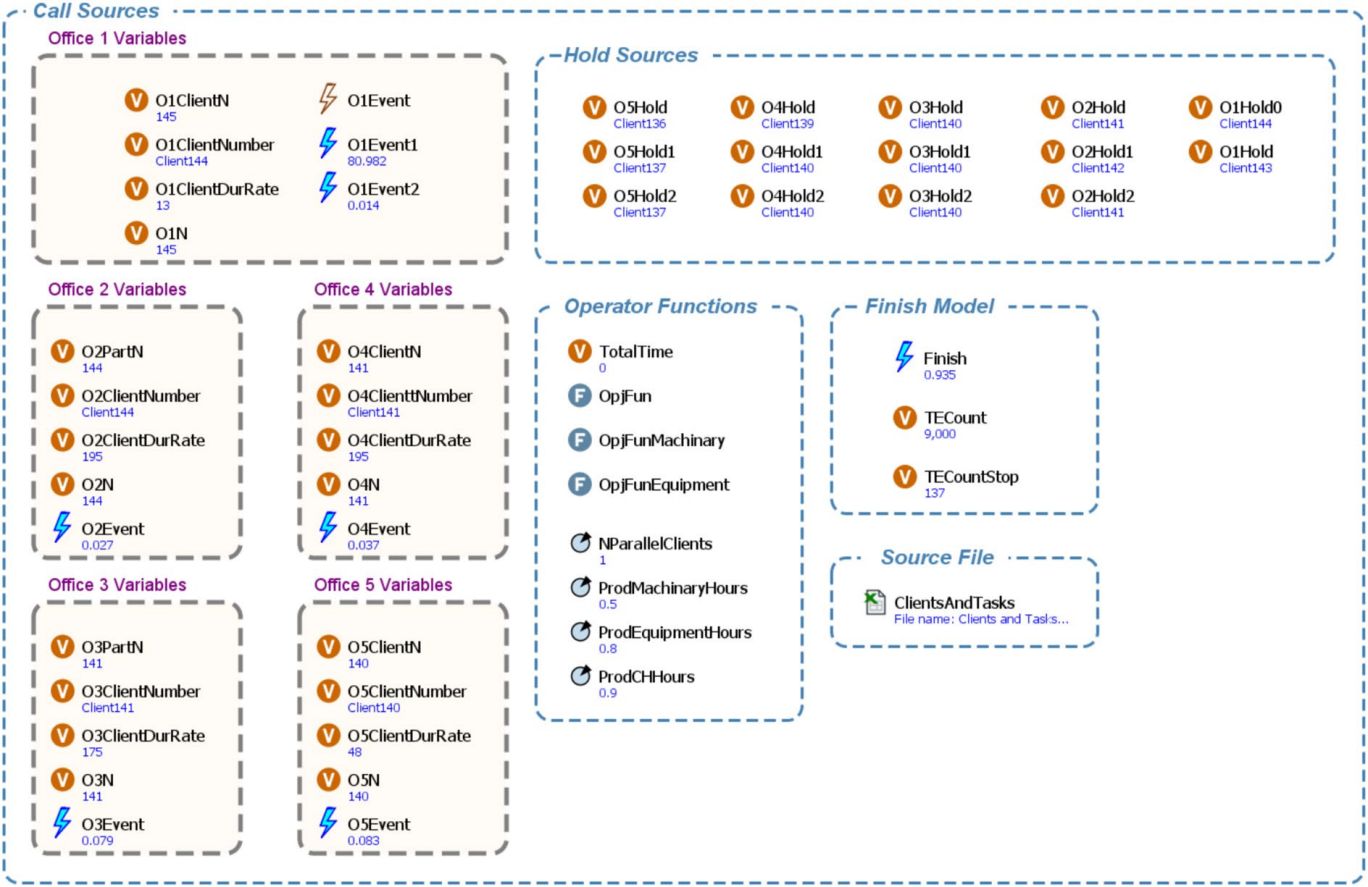
Illustration of the model functions and parameters.

Within the context of the optimization process, our model meticulously evaluates a diverse set of parameters and variables, as shown in (Fig. 5). These metrics include waiting times for elevators, the time required to traverse wheelchair-accessible pathways, and user satisfaction ratings. The quantification of these metrics shows a precise measurement of the building’s effectiveness in serving disabled individuals. Our meticulous attention to modeling queue systems and delays guarantees that the building offers optimal accessibility and convenience to optimize these factors to create an environment that effortlessly caters to the diverse needs of its users. In summation, our model represents a comprehensive and groundbreaking tool that transcends conventional accessibility norms by seamlessly integrating specialized features into the fabric of building design. Through the adept application of advanced simulation and optimization techniques, it not only identifies optimal usage times but also optimizes resource allocation, fostering both inclusivity and efficiency. This innovative approach presents immense value to architects, designers, and policymakers, empowering them to create universally accessible spaces that prioritize the convenience and accessibility of all individuals, regardless of disability.

## Network architecture

Historically, the planning and allocation of resources for the construction of accessible buildings catering to individuals with disabilities often overlooked the specific requirements and features essential for accessibility. Elements such as wheelchair ramps, mobility and sensory support features, hearing aids, and related accommodations were frequently neglected. Consequently, this approach led to the creation of buildings that did not fully comply with accessibility standards, resulting in inconveniences and challenges for individuals with disabilities. The proposed research employs simulation modeling platform to enhance the construction of accessible buildings. This entails developing a comprehensive building network and incorporating elements like delays and queue systems to simulate real-world construction scenarios. Subsequently, the model runs simulations to evaluate the impact of various factors, including resource allocation, construction timelines, and the integration of accessibility features, on the overall efficiency of the building.

Creating the building network from data sources such as architectural plans and construction schedules. These data are translated into a digital representation that incorporates accessibility features. Delays and queue systems are meticulously modeled to simulate construction bottlenecks and resource constraints, providing a realistic portrayal of the construction process. The model has essential functions and parameters for data collection, resource allocation, and time optimization. This includes the ability to dynamically adjust the allocation of resources for tasks related to accessibility feature installation and various construction phases. The model can also consider task dependencies and the availability of specialized equipment for tasks like hearing aid installation or escalator assembly.

Running the simulation yields invaluable insights into the construction of accessible buildings. It furnishes data on the optimal timing for building utilization and the ideal number of resources required to integrate accessibility features efficiently. This information proves indispensable for project planning and management, enabling the cost-effective and timely completion of accessible building projects. Through these comparative analyses, it becomes possible to determine the most efficient balance between construction time and resource utilization to achieve an accessible and well-optimized building. The model operates across three levels, starting with initial simulation runs, then comparative analysis to fine-tune parameters, and culminating in a comprehensive optimization analysis. During the optimization phase, the model endeavors to identify the optimal number of resources required for accessibility feature installation and construction tasks while simultaneously minimizing the overall construction duration. This iterative process ensures that the accessible building not only complies with accessibility standards but is also constructed most efficiently and cost-effectively.

## Results and discussion

The effective scheduling and planning of buildings designed to accommodate disabled individuals, incorporating essential features such as wheelchair accessibility, escalators, and assistive devices such as communication aids, presents a multifaceted challenge. This conclusion outlines vital considerations and emphasizes the critical role of a simulation modeling tool in addressing these challenges. The foremost challenge in designing accessible buildings is acknowledging and accommodating the diverse accessibility needs of disabled individuals, which can be highly individualized. These needs encompass a broad spectrum, ranging from mobility impairments to sensory requirements. Effective building design must consider this variability and strive to provide comprehensive solutions.

In the context of the approach provided in the paper, the elaborate simulation-driven analyses shed light on the intricate dynamics of building accessibility for individuals with disabilities. By leveraging advanced simulation software, such as AnyLogic, the study effectively dissects the multifaceted challenges inherent in optimizing critical elements like wheelchair accessibility, mobility and sensory support features, and assistive devices such as communication aids within building design. These findings align with prior research and underscore the imperative of acknowledging and accommodating the diverse accessibility needs of disabled individuals. The simulation model introduced in this study not only comprehensively examines the utilization of the entire building but also considers the sequence of tasks required for each client, thereby enhancing the realism and applicability of the simulations. Furthermore, the optimization analyses conducted through the model exemplify a meticulous balance between resource allocation and operational timeframes, echoing the findings of previous studies. This optimization process, showcased through iterative numerical runs and numerical optimization techniques, underscores the paramount importance of fine-tuning resource allocations to ensure the efficient operation of buildings tailored to accommodate disabled individuals. The presented results offer valuable insights into optimal usage times and resource allocations, aligning with the overarching goal of promoting inclusivity and user-friendliness within building design.

Moreover, the dynamic presentation of simulation results provides a comprehensive overview of the operational dynamics of inclusive buildings, offering critical insights into the utilization and efficiency of key machinery and equipment items. This level of granularity extends beyond mere machinery statistics, encompassing client service within individual offices and real-time availability of machinery and equipment items, thereby empowering stakeholders to make informed decisions aligned with the evolving needs of individuals with disabilities.

In essence, the results underscore the pivotal role of simulation modeling in optimizing buildings designed for individuals with disabilities, contributing significantly to the advancement of inclusive building design and management practices. These findings not only highlight the importance of leveraging advanced simulation tools but also emphasize the need for continued collaboration among stakeholders to ensure the creation of universally accessible built environments.

### Iterative numerical runs

**Fig. 6:**
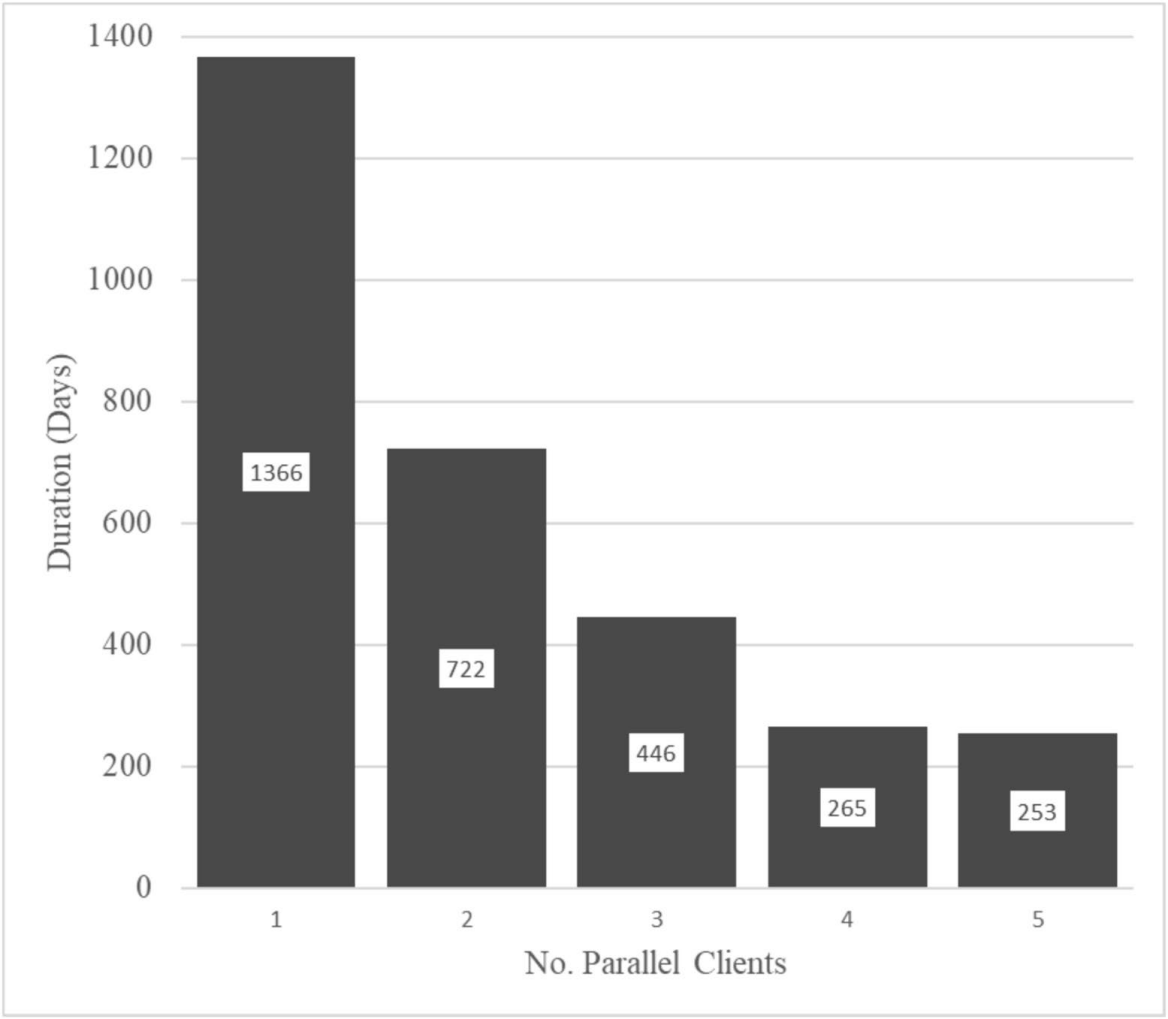
Deterministic Limit approach for the required number of parallel clients.

To ensure the efficient operation of a building designed for disabled individuals, optimizing resource allocation, and determining the most favorable usage times is imperative. Achieving this necessitates an in-depth understanding of the specific needs and preferences of the target user group. Simulation modeling tool offers invaluable capabilities in addressing the challenges of accessibility planning. By constructing a detailed network of the building, replete with elements like delays and queue systems, the developed model facilitates the creation of realistic building’s environment. These models are instrumental in simulating diverse usage patterns, accounting for the movement of disabled individuals throughout the building, potential bottlenecks, and resource demands. Also, the model empowers researchers to compare different scenarios, assessing how variations in construction timelines and resource allocation affect overall efficiency and accessibility, as Illustrated in (Fig. 6).

The results depicted in Fig. 6 provide valuable insights on the impact of varying usage scenarios on building efficiency and accessibility. As illustrated, when considering different numbers of parallel clients utilizing the building simultaneously, notable differences in the duration of service provision are observed. For instance, with a single client, the duration for service provision is 1366 units. However, as the number of parallel clients increases to two, three, four, and five, the duration decreases significantly to 722 (47% decrease), 446 (67% decrease), 265 (81% decrease), and 253 (82% decrease) units, respectively. From this, the user can detect when the change in a certain parameter (Parallel Clients in this case) will become neutral with minor impact. These results underscore the critical importance of optimizing resource allocation and scheduling to accommodate varying levels of usage efficiently. By simulating different usage scenarios, the model allows researchers to assess the impact of resource allocation strategies on overall building efficiency and accessibility. Such insights are invaluable for architects, planners, and policymakers seeking to create buildings that effectively serve the needs of individuals with disabilities while maximizing operational efficiency.

### Numerical optimization

**Fig. 7:**
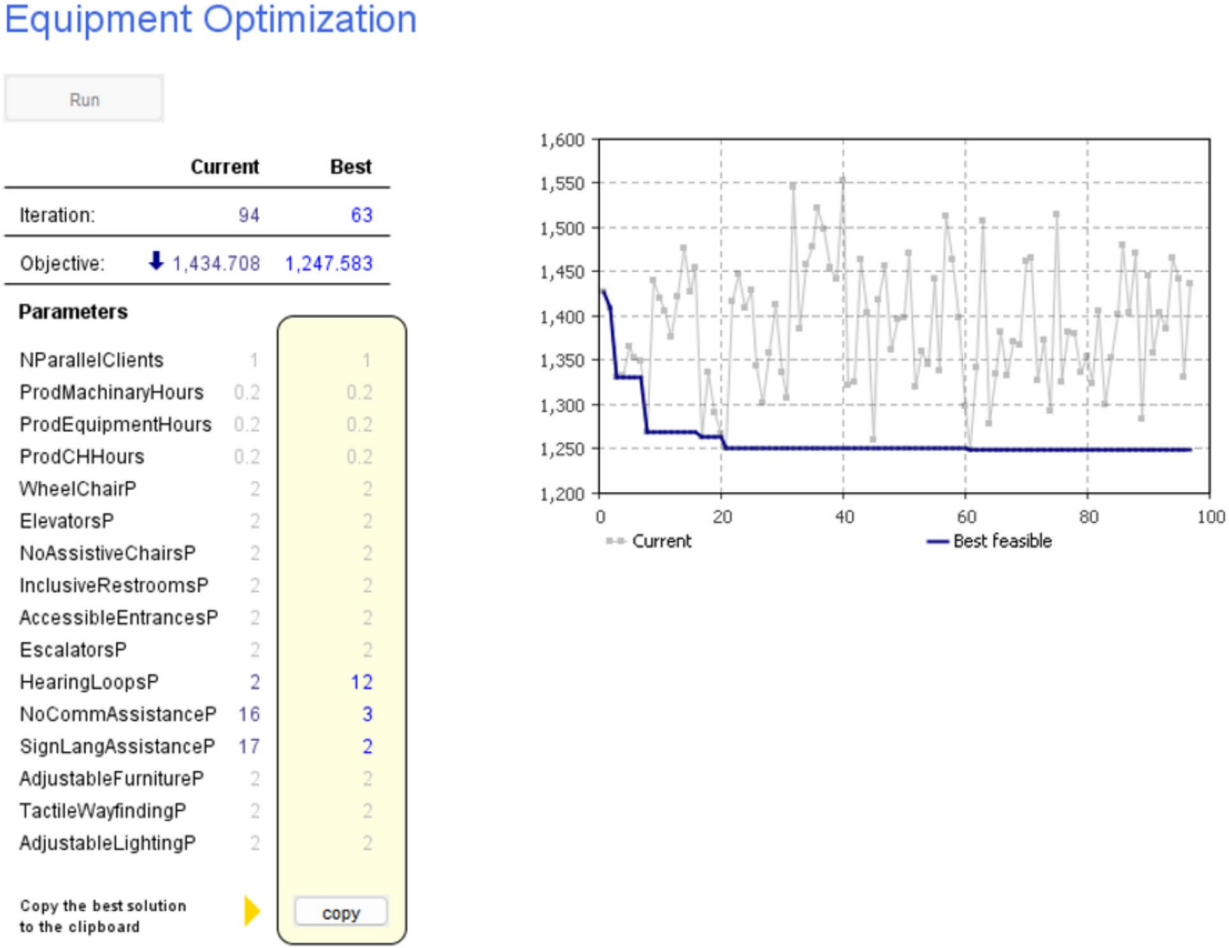
Optimization engine for equipment resources with fixing other parameters.

**Fig. 8:**
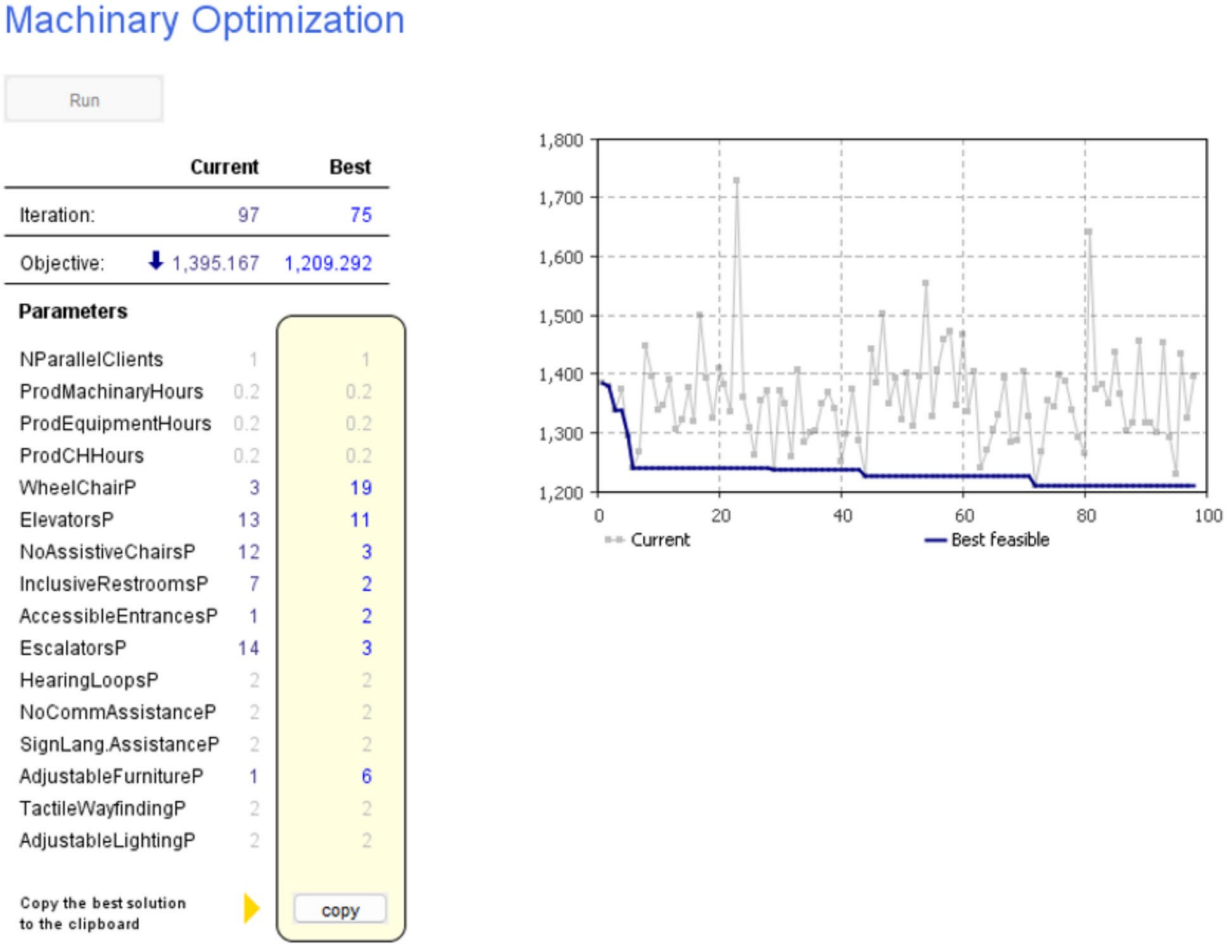
Optimizing machinery resources (e.g. Wheelchairs) while fixing other parameters.

One of the core strengths of the model lies in its ability to conduct optimization analyses, as Illustrated in (Figs. 7, 8). Through this process, the tool assists in determining the ideal allocation of resources, including personnel, equipment, and accessible facilities. By fine-tuning these resource allocations, the building can function efficiently and effectively to meet the needs of disabled individuals. In addition to optimizing resource allocation, this model aids in identifying the most suitable usage times for the building. This involves considering peak and off-peak periods to maximize accessibility and convenience for all users, including disabled individuals. This approach promotes inclusivity and user-friendliness by aligning building usage with user demands. In summary, the utilization of this model in the planning and scheduling of buildings for disabled individuals results in a more inclusive and user-friendly built environment. By addressing the diverse accessibility needs, optimizing resource allocation, and determining optimal usage times, this approach not only enhances accessibility but also optimizes resource utilization and overall building efficiency. It represents a holistic and systematic strategy for creating environments that cater to the needs of all individuals, regardless of their abilities, and exemplifies the importance of leveraging advanced simulation tools in modern building design practices.

One of the core strengths of the model lies in its ability to conduct optimization analyses, as Illustrated in (Figs. 7 and 8). Through this process, the tool assists in determining the ideal allocation of resources, including personnel, equipment, and accessible facilities. Figure 7 demonstrates the model’s effectiveness in optimizing equipment resources by iteratively adjusting their numbers to achieve the most favorable outcomes. After rigorous iterations, the model identified that allocating 12 hearing loops, 3 communication assistance devices, and 2 sign language assistance devices led to a significant optimization in duration, reducing it from 1450 to 1220 days, representing a savings of approximately 15.17%. The logical choice of these numbers stems from a detailed analysis of user needs, building layout, and usage patterns. For instance, while it may seem intuitive to install more communication assistance devices or sign language assistance devices, the model considers factors such as the size of the building, the distribution of users, and the frequency of usage to arrive at the optimal number that ensures adequate coverage without unnecessary redundancy.

Similarly, in Fig. 8, the model optimizes machinery resources to enhance building efficiency and accessibility. The optimization process resulted in the selection of 19 core accessibility elements, 11 elevators, 3 assistive chairs, 2 inclusive restrooms, 2 accessible entrances, 3 escalators, and 6 adjustable furniture units. Again, the logical choice of these numbers reflects a thorough analysis of user needs, building design, and operational requirements. For example, the decision to include only 2 accessible entrances instead of a higher number is based on factors such as traffic flow, proximity to parking areas, and the distribution of entry points to ensure efficient access without overburdening certain areas. Consequently, the duration of service provision decreased from 1400 to 1200 days, reflecting a savings of around 14.29%. By fine-tuning resource allocations and identifying optimal usage times, the model ensures that building operations align with user demands, promoting inclusivity and user-friendliness. In summary, the utilization of this model in the planning and scheduling of buildings for disabled individuals results in a more inclusive and user-friendly built environment. By addressing diverse accessibility needs, optimizing resource allocation, and determining optimal usage times, this approach enhances accessibility, optimizes resource utilization, and improves overall building efficiency. It represents a holistic and systematic strategy for creating environments that cater to the needs of all individuals, regardless of their abilities, and exemplifies the importance of leveraging advanced simulation tools in modern building design practices. The above-mentioned optimization results are due to isolated parameters only while fixing remaining parameters.

### Dynamic optimization and modeling

**Fig. 9:**
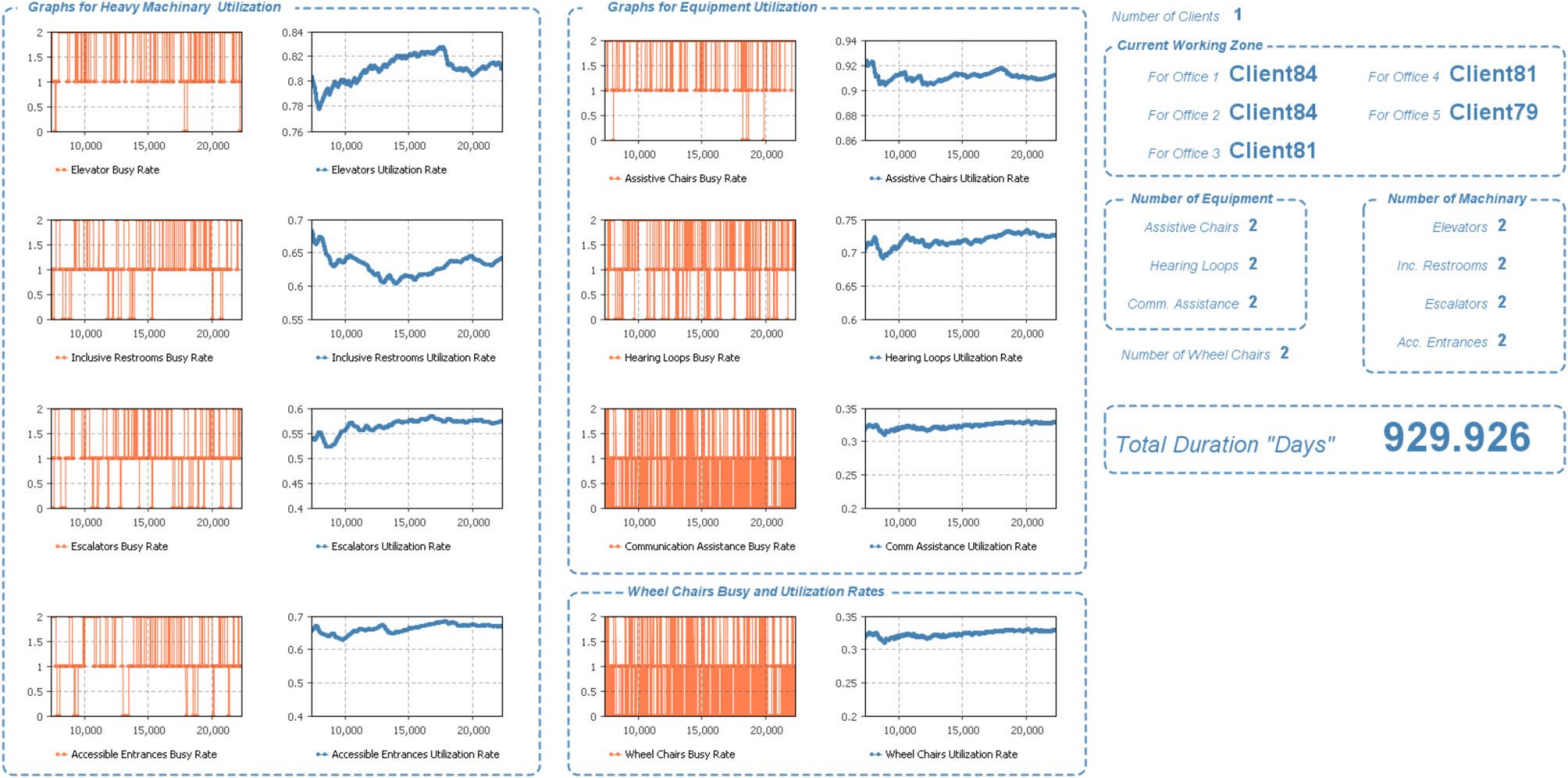
Quantitative charts for results of the model simulation at specific resources case.

Besides the model capabilities to optimize the available resources, the simulation’s results depicted in Fig. 9 showcase the dynamic operational aspects of the inclusive building, revealing critical insights into the utilization and efficiency of key machinery and equipment items. The busy time rates and utilization rates for essential machinery such as elevators, inclusive restrooms, mobility and sensory support features, and accessible entrances are meticulously presented, offering a comprehensive overview of their performance. Similarly, the figure provides detailed information on equipment items, including assistive chairs, hearing loops, communication assistance, and wheelchair availability. The model goes beyond mere machinery statistics, extending its analysis to client service within individual offices, elucidating the number of clients being served concurrently. Moreover, the graph illustrates the real-time availability of machinery and equipment items throughout the entire building during the simulation. One notable highlight of the figure is the emphasis on wheelchair-related data, showcasing the availability and usage patterns. The comprehensive nature of the presented results allows stakeholders to discern patterns in demand and optimize the allocation of resources for improved accessibility. Furthermore, the model facilitates strategic decision-making by indicating the total duration in days required to serve all clients utilizing the inclusive building. This critical metric not only gauges the overall efficiency of the building but also aids in fine-tuning resource allocation and scheduling strategies to minimize delays and enhance the overall accessibility of the facility. Through the optimization of different resource items, the duration decreased significantly from 1450 to 930 days, representing a remarkable savings of approximately 35.86%. It’s worth noting that these efficiency gains translate not only into time savings but also into cost savings, as the reduced duration implies lower operational expenses and a potentially faster return on investment. Overall, the results encapsulated in Fig. 9 provide a holistic understanding of the inclusive building’s operational dynamics, empowering stakeholders to make informed decisions that align with the diverse and evolving needs of individuals with disabilities.

These simulation and optimization result not only validate the modeling framework but also yield significant insights for inclusive building design at both the operational and strategic levels. First, the duration reductions achieved (e.g., from 1450 to 930 days—a 35.86% improvement) illustrate the non-linear benefits of targeted resource allocation. Small increases in accessibility equipment led to large gains in efficiency, confirming the hypothesis that operational performance in disability-friendly infrastructure is highly sensitive to resource distribution. Additionally, the accessibility score improvements observed (e.g., from 72 to 98% across scaling factors) offer actionable metrics for policymakers and facilities managers. This quantifiable scoring system bridges a critical gap in previous literature, where accessibility was often evaluated qualitatively or based solely on code compliance (e.g.^28,40^,). The integration of accessibility score, cost impact, and service time into a unified simulation output introduces a decision-support capability for inclusive planning. Importantly, the study introduces a Resource Investment Efficiency Curve (as seen in Table 1 and Fig. 9), where diminishing returns on ROI are observed beyond 75–80% accessibility improvement. This insight is essential for real-world applications, where resource constraints often limit full compliance with ideal standards. These results show that prioritizing investment in accessibility features up to a calculated efficiency threshold can maximize impact without overextending budgets. This builds upon and extends the cost–benefit concepts discussed in Sherif et al.^31^, where optimization was applied to underground utilities, but now adapted to an inclusive architectural context. Furthermore, the findings affirm that optimization is not only feasible but essential in accessibility-focused design. Rather than assuming that more resources always yield better outcomes, the model offers data-driven guidance on how to balance staff, equipment, and design features. For example, the decision to use only two accessible entrances instead of more was supported by simulation of traffic flow, user distribution, and redundancy analysis—avoiding unnecessary spending while preserving function. Compared to related simulation studies (e.g.^34,35^,), the model presented here is distinguished by its integration of user feedback (via surveys), validation against real buildings in Riyadh, and its focus on operational inclusivity, not just emergency management. This elevates the contribution from a purely theoretical exercise to a practical, transferable framework that can be adapted by municipalities, architects, and infrastructure consultants to enhance inclusive design across public and private sectors.

**Table 1:** Cost-benefit analysis of resource optimization.

Resource	Initial cost (USD)	Optimized cost (USD)	Savings (USD)
Core accessibility elements	15000	19000	−4000
Elevators	80000	88000	−8000
Assistive chairs	3000	2500	500
Inclusive restrooms	2000	1800	200
Accessible entrances	3000	2800	200
Escalators	15000	12000	3000
Adjustable furniture	7000	5000	2000

Beyond technical performance, the success of any inclusive design model must be evaluated through the lens of equity, representation, and participatory ethics. While this study integrates feedback from survey responses of individuals with disabilities, future development phases must prioritize co-design practices, where people with disabilities are engaged as collaborators rather than passive data sources. Their lived experiences offer critical insights that often escape purely technical models—such as spatial anxiety, communication friction, or psychological stress related to environmental unpredictability. Participatory modeling frameworks, such as those advocated by Hofmann et al.^33^ and Terashima and Clark^2^, emphasize the importance of including disabled stakeholders in the framing of research questions, prioritization of metrics, and validation of results. For example, the accessibility “score” used in this simulation could be refined through focus group discussions, ensuring that the metric reflects not only quantitative resource availability but also qualitative user satisfaction. From a disability justice standpoint, it is essential to acknowledge that not all impairments can be addressed through optimization alone. The model’s assumptions currently focus on physical access and assistive technologies, but future versions must expand to account for neurodiversity, invisible disabilities, and intersectional identities (e.g., age, gender, income) that shape how disability is experienced in different social and architectural contexts. Additionally, ethical modeling should avoid framing disability as a problem to be solved solely through efficiency. Instead, it should aim to build adaptive environments that respect autonomy, variability, and the right to access with dignity. Embedding this philosophy in the simulation process means redefining performance indicators not just in terms of time or cost saved, but also in terms of empowerment, freedom of choice, and social inclusion. Therefore, this study proposes that future iterations of the model adopt a participatory design protocol throughout the simulation lifecycle—from agent definition and scenario setup to validation and output interpretation. This would align the work more closely with universal design principles, ensuring that optimization strategies support not only operational efficiency but also human rights and inclusion.

### Quantitative statistical analysis

**Fig. 10:**
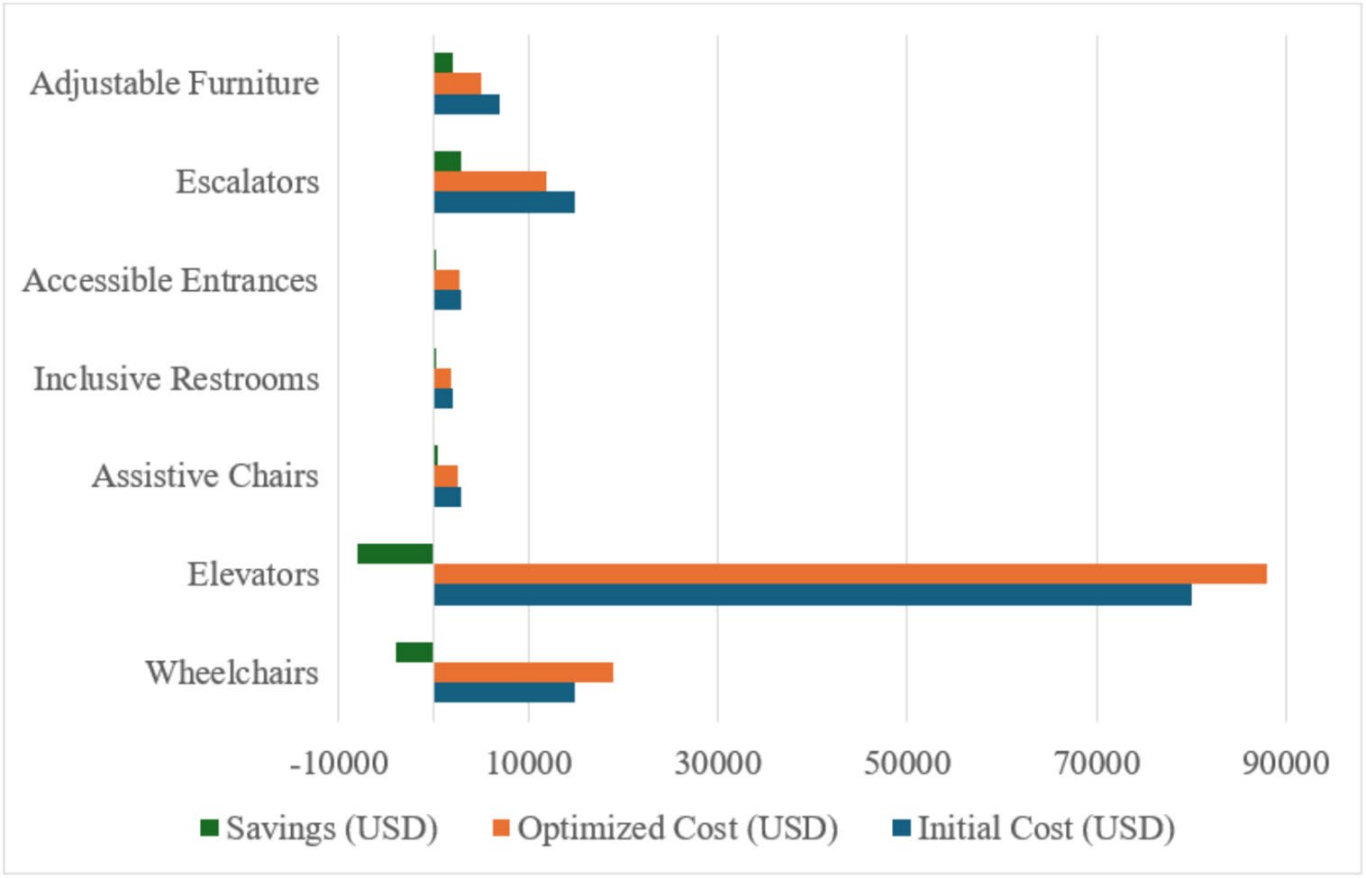
Cost-benefit analysis of resource optimization Comparison.

The cost-benefit analysis of resource optimization highlights the financial implications of adjusting resource allocations for improved accessibility. The findings presented in Table 1 and Fig. 10 reveal that while certain resources, such as elevators and core accessibility elements, required increased expenditure due to higher quantities, other resources, such as assistive chairs and adjustable furniture, demonstrated cost savings. For instance, the optimized allocation of adjustable furniture reduced costs by USD 2000, whereas the additional elevators increased costs by USD 8000. These results emphasize the trade-offs inherent in optimization, where enhancing accessibility often necessitates increased investments in critical resources. The analysis underscores the need for a strategic approach to resource allocation that balances cost considerations with the imperative to meet accessibility standards effectively. Decision-makers can leverage these insights to prioritize resource investments that deliver the highest utility for individuals with disabilities while maintaining financial sustainability.

Also, it is important to mention that All cost values (e.g., elevators, assistive chairs, hearing loops) were obtained from local vendor quotes in Saudi Arabia and cross-referenced with procurement data from accessibility retrofit projects in Riyadh. Costs include installation, calibration, and expected maintenance over 5 years^41–43^.

**Fig. 11:**
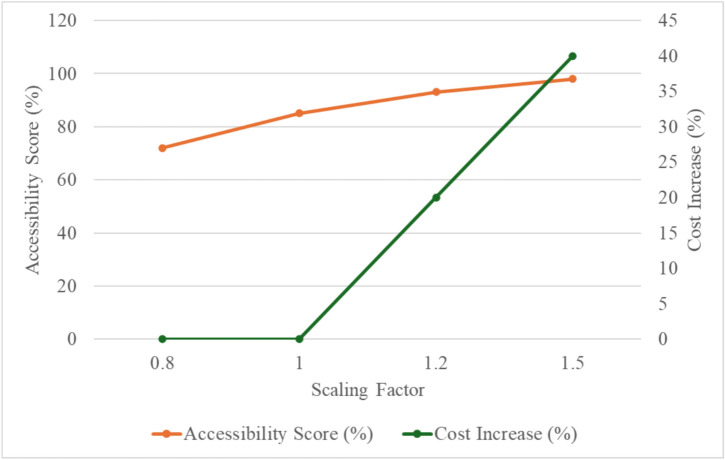
Impact analysis of resource scalability and comparison.

The impact analysis of resource scalability as shown in Table 2 and Fig. 11 demonstrates the effects of varying resource allocations on service duration, accessibility scores, and associated costs. Scaling down resource allocations by 20% (scaling factor 0.8) resulted in a significant increase in service duration to 1500 units and a corresponding drop in accessibility scores to 72%. Conversely, scaling up by 50% (scaling factor 1.5) achieved an accessibility score of 98% and reduced service duration to 900 units, albeit with a 40% increase in costs. These findings indicate a clear trade-off between cost and accessibility outcomes, with diminishing returns observed as resource quantities exceed the baseline allocation. This analysis emphasizes the importance of determining an optimal scaling factor that achieves high accessibility scores without disproportionately escalating costs, providing a roadmap for resource management in the design and operation of inclusive buildings.

**Table 2:** Impact analysis of resource scalability.

Scaling factor	Service duration (units)	Accessibility score (%)	Cost increase (%)
0.8	1500	72	0
1	1300	85	0
1.2	1100	93	20
1.5	900	98	40

**Fig. 12:**
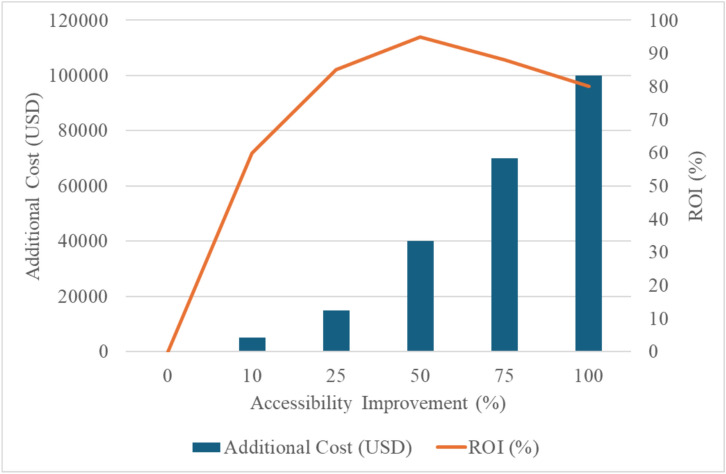
Trade-off analysis of accessibility resources significance.

Also, the trade-off analysis as summarized in Table 3 and Fig. 12 between accessibility improvements and cost increments provides critical insights into optimizing accessibility outcomes. As accessibility improves, the associated costs rise, with the (Return On Investment) ROI peaking at 95% for a 50% accessibility improvement. Beyond this threshold, the ROI begins to decline, with 100% accessibility requiring USD 100,000 in additional costs but yielding an ROI of 80%. This analysis highlights the challenges of achieving full accessibility, where costs escalate significantly with only marginal returns. The findings suggest that prioritizing accessibility improvements up to 50–75% represents the most efficient use of resources, ensuring meaningful enhancements in inclusivity while maintaining cost-effectiveness. These insights enable stakeholders to make informed decisions when balancing accessibility objectives with financial constraints, aligning design strategies with the overarching goals of equity and sustainability.

**Table 3:** Trade-off analysis of accessibility resources.

Accessibility improvement (%)	Additional cost (USD)	ROI (%)
0	0	0
10	5000	60
25	15,000	85
50	40,000	95
75	70,000	88
100	100,000	80

## Conclusion

Simulation models play a pivotal role in optimizing buildings designed to accommodate individuals with disabilities, encompassing features such as wheelchair accessibility, mobility and sensory support features, and assistive devices such as communication aids. These models serve as instrumental tools for evaluating the utilization of such buildings by simulating various scenarios and assessing associated resource requirements and timeframes. The incorporation of simulation modeling techniques within the framework of this paper marks a pivotal stride forward in the realm of inclusive building design. By employing sophisticated simulation software, the study delves deep into the complexities of optimizing structures to accommodate individuals with disabilities. Through meticulous analysis and refinement facilitated by simulation, the research contributes substantially to enhancing accessibility and operational efficiency within such buildings. The modeling process involves creating a detailed network of the building, complete with delays and queue systems, to accurately simulate real-world conditions. Running multiple scenarios allows for the accounting of various usage patterns and demands, providing valuable insights into building utilization. Subsequently, optimization analyses are conducted to determine the optimal number of resources and the time necessary for the building’s optimal operation. Furthermore, the application of simulation modeling in building design has demonstrated significant productivity improvements, with the model achieving savings in productivity levels. These findings underscore the effectiveness of simulation models in optimizing building design and management, further emphasizing their importance in creating inclusive and efficient built environments.

In summary, through the optimization of different resource items, the duration decreased significantly from 1450 to 930 days, representing a remarkable savings of approximately 35.86%. These efficiency gains translate not only into time savings but also into cost savings, as the reduced duration implies lower operational expenses and potentially faster return on investment. Moving forward, it is recommended to continue investing in simulation modeling tools and research to advance building design and management practices. Collaboration among architects, planners, policymakers, and individuals with disabilities is essential to ensure that simulation models accurately reflect diverse needs and preferences. Additionally, incorporating simulation modeling into building codes and regulations can promote the development of universally accessible built environments. Providing training and education programs for professionals in the architecture and construction industries will enable them to effectively utilize simulation modeling for inclusive building design. Furthermore, awareness campaigns are needed to highlight the significance of simulation modeling in creating inclusive and efficient buildings and its positive impact on society. These recommendations and implications underscore the importance of leveraging simulation modeling to drive progress in inclusive building design and management.

This study acknowledges that inclusive design is not solely a technical endeavor. The model’s development was informed by survey responses from disabled users, yet further co-design sessions and participatory modeling practices are essential. Future iterations should incorporate lived experience narratives to guide parameter prioritization and validation processes. Embedding equity and disability justice principles into simulation modeling strengthens both ethical alignment and practical relevance.

## Data Availability

Data supporting this study’s findings are available from the corresponding author upon reasonable request.
